# Cross-sectoral collaborations and funding and coordination mechanisms in One Health zoonoses management in Peru

**DOI:** 10.3389/fpubh.2026.1799546

**Published:** 2026-05-06

**Authors:** Lisset Dumet Poma, Erin S. Kenzie, Julia Goodman, Veronika Merino, Vanessa Cruz, Ruth Atto, Percy Vilchez, Seth O’Neal

**Affiliations:** 1 Chestnut Health Systems – The Lighthouse Institute, Eugene, OR, United States; 2 School of Public Health, Oregon Health & Science University – Portland State University, Portland, OR, United States; 3 Oregon Institute of Occupational Health Sciences, Oregon Health and Science University, Portland, OR, United States; 4 Cayetano Heredia Peruvian University, Lima, Peru; 5 Center for Global Health, Tumbes, Peru; 6 Prisma, Lima, Peru

**Keywords:** low-resource settings, multiple-methods, multisectoral collaborations, One Health, rapid-qualitative method, social network analysis, zoonoses

## Abstract

One Health (OH) is a comprehensive approach that recognizes the human-animal-environment interconnection to health and is applied to prevent and control zoonoses—diseases transmitted between animals and people. Using a multiple-method case study, we examined zoonoses networks, resource management mechanisms, and coordination strategies among policymakers and decision-makers of Peru’s human, agricultural, and environmental systems at national and sub-national levels. Social network analysis revealed collaborations between the human and agricultural systems, with limited connection with environmental systems. Only a few links were reported between national and sub-national government levels. Qualitative analysis identified structural barriers, including insufficient regulatory mechanisms for funding cross-sectoral activities. Public financing structures created siloes and resource disparities across systems, hindering sustained multisectoral collaboration. There is a need to regulate the role of environmental systems, including forest protection services, in OH initiatives. Local regulations were used to formalize work agreements with local organizations and compel participation in multisectoral activities. A nationwide OH policy that mandates multisectoral engagement and coordination is needed, as are mechanisms to engage and empower local authorities, community leaders, and farmers in local surveillance systems. Future policy research should assess the evolution of OH policy networks to inform sustainable collaboration strategies.

## 1 Introduction

Zoonoses, diseases transmitted between animals and people, inflict a heavy toll on the economy, health, and livelihood worldwide and disproportionately affect low-resource countries (1, 2). The prevention and control of zoonotic diseases require comprehensive approaches, such as One Health (OH), that promote collaboration, communication, and coordination among disciplines and sectors, including local communities and governments, to improve the health of humans, animals, and plants (3). OH research and interventions require applying bottom-up strategies—developed at the community or local level—to understand local organizational systems and community needs (4). However, in low-resource countries (LRC) characterized by hierarchical organizational systems, it is also essential to study top-level policy determinants, such as policy networks (5). Understanding the structure of policy networks and how they interact will provide essential information on the roles of interested parties and the interconnections among systems, informing future OH multisectoral collaborations (6, 7).

Recommendations to enable OH multisectoral collaborations propose studying initial policy process conditions using systems-thinking methods to examine organizational networks and administrative rules that support partnership building (8, 9). Social network analysis (SNA) is a systems science approach for studying cross-sectoral networks (10). SNA has been used to study homophily in OH networks, which posits that individuals with similar personal traits, such as discipline or organizational affiliation, are more likely to connect (11).

Studies analyzing semi-structured interviews with OH initiative participants in India and Mexico have found that OH continues to exhibit high homophily in stakeholder collaborations, reporting more connections with peers within their organization than with those outside it (12, 13). The authors conclude that SNA is needed to evaluate OH collaboration outcomes and to include sub-national governmental levels and non-governmental institutions. Our paper addresses this research gap by employing a multiple-methods approach combining SNA and the rapid qualitative method (RQM) to examine the policy networks of national and sub-national systems involved in preventing and controlling zoonoses in Peru.

### 1.1 Peruvian zoonoses prevention and control landscape

**Figure 1:**
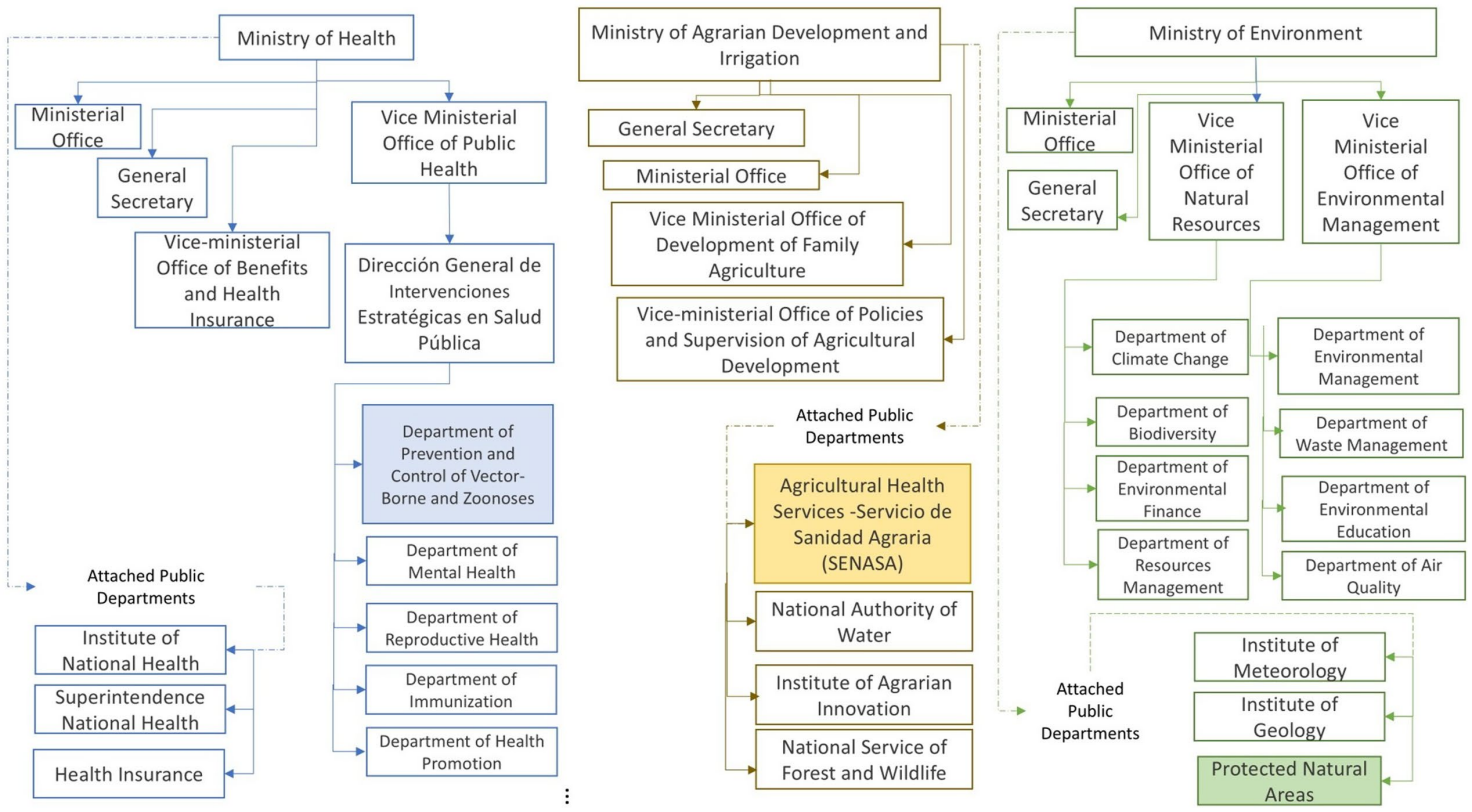
Organizational chart for the Ministries of Health, Agriculture, and Environment.

Peru has a decentralized government that endowed the sub-national level, regions, provinces, and districts with political, economic, and administrative autonomy (14). The regional governments oversee local public institutions and exercise administrative, economic, and political power and autonomy. The regional government develops and approves regional norms and policies, designs, manages, and implements projects (15). Figure 1 presents the country’s organizational chart for the Ministries of Health, Agriculture, and Environment, highlighting the units responsible for zoonoses management. The Peruvian government unit coordinating strategic public health interventions to address zoonoses is the Department of Prevention and Control of Vector-Borne Diseases and Zoonoses, part of the Peruvian Ministry of Health. This unit follows the directives of the Vice-Ministerial Office of Public Health. The unit responsible for managing animal health, mainly livestock, is the Agricultural Health Services, and the unit responsible for managing forest and wildlife is the National Forestry and Wildlife Service, both within the Ministry of Agriculture.

In 2019, a national policy was passed to create a multisectoral commission for the prevention and control of zoonotic diseases, to propose policies and guidelines for managing zoonoses, and to establish guidelines to promote multisectoral collaboration among various ministries (16). Another multisectoral initiative is a temporary working group on surveillance, prevention, and control of Avian Influenza to mitigate the risk of transmission from animals to humans, comprising national and sub-national multisectoral organizations (17).

The Peruvian landscape provides an ideal case study for assessing cross-sectoral networks. We expect to see a network of multisectoral connections among zoonotic disease systems, particularly between the Ministries of Health and Agriculture. However, based on previous literature and the absence of formal OH implementation in Peru, we hypothesize a high level of homophily in zoonoses collaborations (12, 13).

### 1.2 Theoretical background and research aims

The Institutional Analysis and Development (IAD) Framework for policy analysis (18), the Social-Ecological Systems (SES), and the One Health framework inform the conceptual framework in our research, depicted in a figure in Supplementary material 1 (3, 19, 20). This study is part of a broader policy analysis series that examines the community attributes, conditions, rules-in-use, and interactions to inform OH collaborations (21–23). In this paper, we propose two research objectives: (1) Determine the network structure assessing multisectoral interactions within and among national and sub-national organizations involved in multisectoral collaborations to prevent and control zoonoses; and (2) Describe the mechanisms for sharing resources and coordination in OH multisectoral collaborations.

## 2 Materials and methods

### 2.1 Ethics approval

All research participants signed a consent form before participating in the study. Ethical approval was obtained from Oregon Health and Science University (IRB ID: STUDY00024870) and Cayetano Heredia University (IRB ID: 209853).

### 2.2 Study methodology

We applied a multiple-methods approach combining social network analysis (SNA) and the rapid qualitative method (RQM) to examine multisectoral collaborations for zoonotic disease prevention and control in Peru. We included national organizations—primarily the Ministries of Health, Agriculture, and Environment—and sub-national agencies in Tumbes and Piura. Participants were purposively and snowball-sampled policymakers and experts from human, animal, and environmental sectors. Data were collected from June to November 2023 through network surveys and semi-structured interviews, yielding 77 surveys and 75 interviews. Interviews were conducted mainly in person, in Spanish, recorded with consent, and transcribed with identifiers removed to ensure anonymity.

Network survey questions captured collaboration patterns, leadership nominations, and cross-organizational ties. Using RStudio and UCINET, we calculated in-degree and out-degree multidisciplinary or multisectoral connections by organization and the percentage of connections within and outside organizations. We calculated homophily using the homophily test on the entire network partitioned by organization affiliation (24, 25). Semi-structured interviews explored policy processes, multisectoral collaboration experiences, and mechanisms for resource sharing and accountability. Using RQM, two coders iteratively summarized and synthesized transcripts into matrices to identify patterns across ministries and government levels (26). Themes were generated through independent reviews and comparison of condensed summaries to characterize similarities and differences in zoonoses management across organizational groups. A more detailed description of data collection, measures, and analysis, as well as network survey questions and the interview guide, is provided in Supplementary material 2.

## 3 Results

A total of 77 participants completed the network survey, and 75 completed the semi-structured interview. We excluded two incomplete interviews: one with a participant affiliated with the Ministry of Health at the national level and one with a participant who was either a regional authority or a municipal administrator at the sub-national level. Organizations’ affiliations were 47% at the national level, 40% at the sub-national level, and 13% in external organizations. Within the national tier, the Ministry of Health accounted for the largest share (~72%), followed by the Ministry of Agriculture (~22%) and the Ministry of Environment (~6%). At the sub-national tier, Regional Directorates of Health account for the majority (~61%), followed by Regional Directorates of Agriculture (~16%), and regional/municipal authorities contribute ~23%. Among external organizations, academia and education make up the bulk (80%), and international organizations comprise the remaining 20%.

### 3.1 Description of zoonoses policy networks

**Figure 2:**
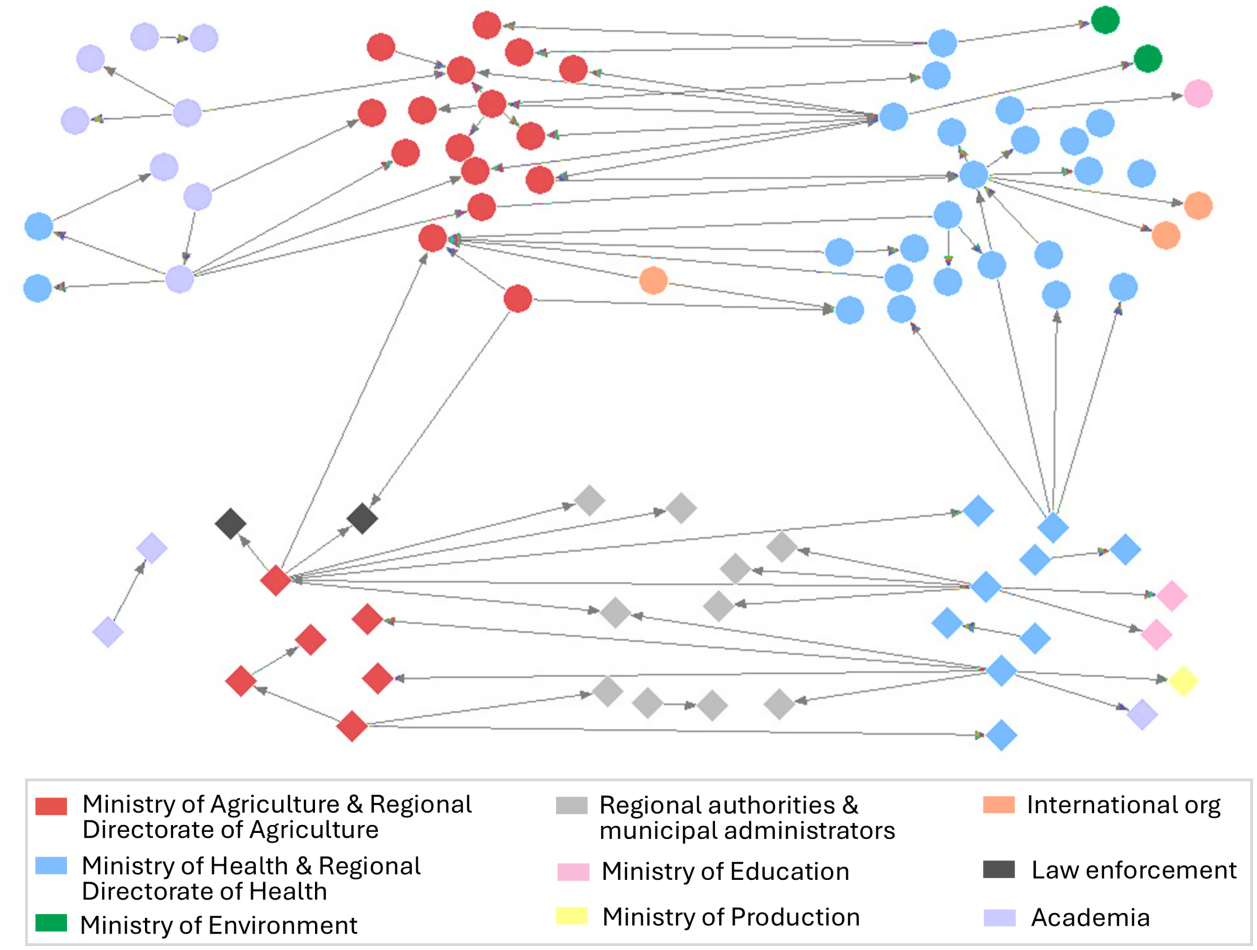
Social network diagram showing multisectoral connections among the various systems involved in the prevention and control of zoonoses in Peru. Nodes at the top and with a circular shape represent national-level responders, and nodes at the lower level with a diamond shape represent the sub-national level.

Figure 2 displays participants’ organizational affiliations and the individuals with whom they have worked in multisectoral or multidisciplinary collaborations across Peru’s national and sub-national systems to prevent and control zoonoses.

Figure 2 highlights the few connections reported between participants who belong to sub-national and national levels (six nominations in total). Most of these nominations were made by participants affiliated with the Ministry of Health, specifically from one participant representative of the sub-national level to four others at the national governmental level. The remainder were from representatives of the Ministry of Agriculture at the national level, who either nominated or received nominations from sub-national level affiliated with law enforcement organizations.

SNA revealed that, on average, each participant reported working with one person in zoonoses multisectoral or multidisciplinary collaboration. Results also show several multisectoral connections among participants affiliated with organizations outside the animal-human-environment system’s boundaries, such as the Ministries of Education and Production. At the national level, we observe more connections between participants affiliated with the Ministry of Health and those affiliated with the Ministry of Agriculture. In contrast, at the sub-national level, regional authorities and municipal administrators are essential in zoonoses multisectoral collaborations connecting the human and animal sectors or working with either of them.

Table 1 includes SNA measures that quantify the connections shown in Figure 2: out-degree, the number of nominations given, and in-degree, the number of nominations received, for zoonoses multisectoral or multidisciplinary collaborations organized by sector. The top three organizations with the highest out-degree, or those that reported the most multisectoral or multidisciplinary collaborations, were the Ministry of Health, the Regional Directorate of Health, and the Ministry of Agriculture (24, 17, and 14 nominations, respectively, out of a total of 81). Table 1 also presents additional details on the frequencies and percentages of out-degree by organization. For instance, the Ministry of Health participants reported having worked in total with 24 people in multisectoral collaborations, of which 46% of these collaborations were with others affiliated with the Ministry of Agriculture, 29% with others within their own organization, 8% with the Ministry of Environment, 8% with international organizations, 4% with the Ministry of Education, and 4% with Academia and Education.

**Table 1:** Outdegree (nominations given) and indegree (nominations received) of multidisciplinary or multisectoral collaborations by organizations.

Organizations	Ministry of Health		Ministry of Agriculture		Ministry of Environment		Ministry of Education		Ministry of Production		Regional Directorate of Health		Regional Directorate of Agriculture		Regional Directorate of Education		Regional authority/municipal administrator		Academia & Education		Law enforcement institutions		International organizations		Outdegree: total of nominations given
	*n*	%^b^	*n*	%^b^	*n*	%^b^	*n*	%^b^	*n*	%^b^	*n*	%^b^	*n*	%^b^	*n*	%^b^	*n*	%^b^	*n*	%^b^	*n*	%^b^	*n*	%^b^	
Ministry of Health	*n*	7	35%	11	48%	2	100%	1	100%											1	14%			2	100%	24
	%^a^		29%		46%		8%		4%												4%				8%	
Regional Directorate of Health	*n*	4	20%	1	4%					1	100%	2	50%	2	33%	1	100%	5	45%	1	14%					17
	%^a^		24%		6%						6%		12%		12%		6%		29%		6%					
Ministry of Agriculture	*n*	6	30%	4	17%									2	33%			1	9%			1	33%			14
	%^a^		43%		29%										14%				7%				7%			
Regional Directorate of Agriculture	*n*			1	4%							2	50%	2	33%			4	36%			2	67%			11
	%^a^				9%								18%		18%				36%				18%			
Academia & Education	*n*	2	10%	5	22%															5	71%					12
	%^a^		17%		42%																42%					
Regional authority/municipal administrator	*n*																	1	9%							1
	%^a^																		100%							
International organizations	*n*	1	5%	1	4%																					2
	%^a^		50%		50%																					
Indegree: total of nominations received		20		23		2		1		1		4		6		1		11		7		3		2		81

a Percentage of outdegree by organization.

b Percentages of indegree by organization.

The three organizations that received the most nominations for multisectoral collaboration from other participants, or the highest in-degree, were the Ministry of Agriculture, the Ministry of Health, and the regional authority or municipal administration (23, 20, and 11 nominations, respectively, out of a total of 81). The percentages of in-degree by organization indicate that the Ministry of Agriculture received the highest number of nominations for multisectoral collaborations from participants across organizations. Of the 23 nominations they received, 48% were from participants from the Ministry of Health. In contrast, the Ministry of Health received 20 nominations, with the majority (35%) from participants within its organization, followed by the Ministry of Agriculture (30%).

Results from the homophily test, which assesses group differences in a tie, indicated a Pearson’s chi-square statistic of 156.931 (*p* < 0.0002), rejecting the homophily hypothesis. That is, the expected connection frequencies between members of the same organization are lower than those among different organizations.

### 3.2 Mechanisms for sharing resources and coordination in multisectoral collaborations

From RQM results, we learned that most participants reported participating in multisectoral collaboration to prevent and control zoonoses. One of the programs mentioned was the Multisectoral Commission for the Prevention and Control of Zoonotic Diseases, a collaboration between the Ministry of Health and the Ministry of Agriculture (16). At the sub-national level, respondents indicated participating in multisectoral periodic regional meetings to discuss regional problems, including zoonoses such as rabies and avian influenza. Most participants were familiar with OH, which was applied in a zoonoses prioritization workshop organized by an international organization. Participants believed the application of OH incentivized the inclusion of the environmental sector in zoonotic multi-sector collaborations.

**Figure 3:**
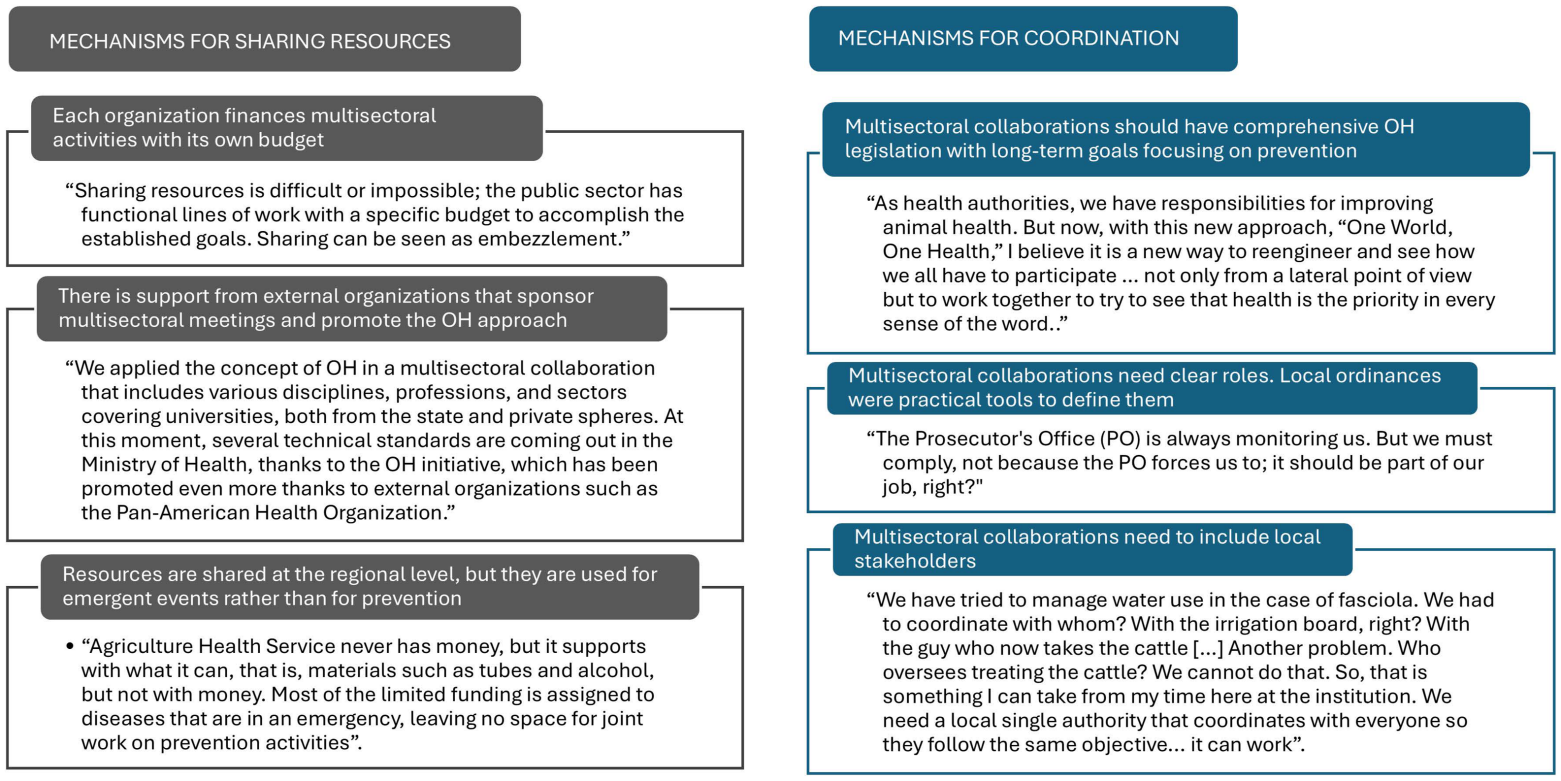
Main themes and quotes regarding resource sharing and coordination mechanisms for OH multisectoral collaborations.

Figure 3 highlights relevant quotations and central themes regarding resource sharing and coordination mechanisms for collaborative multisectoral activities. Supplementary material 3 includes an extended table and a detailed description of the central themes and participants’ experiences in multisectoral collaboration, organized by organization.

#### 3.2.1 Mechanisms for sharing resources in multisectoral collaborations

Participants from various ministries reported that each organization finances multisectoral activities with its own budget to build human capacity, provide laboratory support, or provide transportation to complete them. According to participants in the Ministry of Health, multisectoral activities were coordinated based on their organizational and program functions. Participants reported that resource sharing is highly complex because regulations and public financing structures do not permit funds to be shared among ministries. They explained: “Sharing resources is difficult or impossible; the public sector has functional lines of work with a specific budget to accomplish the established goals. Sharing can be seen as embezzlement.”

At the sub-national level, participants reported that some information is shared between the health and agriculture departments; however, there is no consistency, resulting in delays, duplication, and waste because there are no clear rules governing what can be shared. One participant expanded on this theme regarding multisectoral activities: “Each one went with their own resources and vans. If we had worked in a coordinated manner, we could go together, save time, and share information, but in my experience, each works in isolation.”

Participants reported receiving support from external organizations to sponsor multisectoral meetings and promote the OH approach. The Ministry of Health was reported to be the primary funder of regional meetings and coordination activities within multisectoral collaborations to prevent and control zoonoses. International organizations such as the Pan American Health Organization (PAHO) were cited as the primary sponsors of multisectoral meetings, providing grants to coordinate or finance them. Non-governmental organizations (NGOs) and research organizations were considered promoters of multisectoral activities. A participant from the human health sector described how international organizations helped improve surveillance of infectious diseases: “PAHO, for example, has an initiative for influenza control, and it has to do, obviously, with ‘One Health’ […] it is an international network of respiratory viruses that served to exchange information on coronavirus and now influenza genomics.”

Participants in the Ministry of Agriculture reported receiving financial support from producers and private institutions to respond to zoonotic emergencies, such as Avian Influenza. For instance, because the Forest and Wildlife Management lacks laboratories for diagnosing zoonoses in wildlife, it received support from universities and private laboratories to test infected wild birds and mammals.

Participants reported that resources are shared at the regional level. Although public financing structures do not permit fund sharing, representatives from the human and agricultural sectors reported sharing equipment and information during multisectoral activities at the regional level. Additionally, they mentioned that municipalities contributed to zoonosis prevention efforts by funding activities such as garbage collection, health promotion, and slaughterhouse supervision. One participant from the human health sector said, “Allies such as municipalities are sought to supplement the budget, although this support is often denied.” Participants in the agriculture sector reported that the primary barrier to completing multisectoral activities was limited resources. They said: “Agriculture Health Service never has money, but it supports with what it can, that is, materials such as tubes and alcohol, but not with money. Most of the limited funding is assigned to diseases that are in an emergency, leaving no space for joint work on prevention activities.”

#### 3.2.2 Mechanisms for coordination in multisectoral collaborations

Participants believed that multisectoral collaborations should include comprehensive OH legislation with long-term goals focused on prevention rather than emergencies. They said legal mechanisms, such as ministerial resolutions, could be useful tools for assigning roles to members from different sectors and for agreeing on a long-term strategy that is currently missing. Respondents added that an OH application was essential in multisectoral collaborations. One said: “We know that zoonoses are multi-factorial, in which different ministries must participate; that is why we need to understand the problem and who can address it according to their functions.” However, they identified several barriers to OH implementation, including production-oriented animal health systems that support wildlife domestication. Political instability was also reported as a barrier to OH sustainability, with high turnover hindering meaningful interactions and trust-building in collaborations.

Participants thought that multisectoral collaborations need clear roles. Local ordinances were practical tools to define them. They said it is essential to discuss functions and competencies among experts to determine responsibilities in multisectoral collaborations and to define the roles of sectors that still need to be included in zoonoses prevention and control, such as the Ministry of Environment. Ministry of Agriculture participants at the national and sub-national levels believed that clear rules for the leader selection process, meeting frequency, and penalties for noncompliance were essential, as these could affect trust. Participants described how some meeting agreements sometimes do not translate into activities or improvement: “Sometimes it is a ‘dead letter’, meetings are held, everyone participates, but in the end, it remains in the document, lacking real work and commitment.” Establishing clear incentives for participation, such as publication in academic journals, was also recommended to ensure the work’s continued progress.

Participants described local legal mechanisms to compel participation with a possible penalty for not fulfilling responsibilities: “The Prosecutor’s Office (PO) is always monitoring us. But we must comply, not because the PO forces us to; it should be part of our job, right?” They also described local ordinances as helpful tools for implementing measures to control infectious diseases, including fines for communities that evade participation. Local ordinances also include detailed joint work agreements among local organizations with close ties to community members, such as schools and churches.

Multisectoral collaborations need to include local stakeholders. Respondents reported that regional leaders, such as the regional governor and mayors, should lead multisectoral collaborations to guarantee the participation of relevant actors. Community leaders’ and producers’ participation was essential to ensure sustainability and fairness. Producers were reported to advocate for fair solutions in zoonosis control and prevention, such as agricultural insurance to provide economic compensation in the event of livestock culling. Participants described the complexity of coordinating many local actors in zoonoses multisectoral activities, such as fasciola, an infectious disease transmitted by sheep and cattle to humans through contaminated water: “We have tried to manage water use in the case of fasciola. We had to coordinate with whom? With the irrigation board, right? With the guy who now takes the cattle […] Another problem. Who oversees treating the cattle? We cannot do that. So, that is something I can take from my time here at the institution. We need a local single authority that coordinates with everyone so they follow the same objective… it can work.”

## 4 Discussion

We applied multiple methods, SNA and RQM, to study policy networks among the human, animal, and environmental systems responsible for zoonosis prevention and control at Peru’s national and sub-national levels of government. SNA results show several multisectoral connections, mainly between participants affiliated with the human and agriculture sectors, with the regional authority or municipal administrators also playing an essential role in zoonoses multisectoral collaborations at the sub-national level. Contrary to the latest research reporting high homophily in OH multisectoral collaborations in India and Mexico, we did not find support for the homophily hypothesis in our SNA-based study, meaning connections among participants affiliated with different organizations were more likely to be reported than those within the same organization (12, 13).

Homophily suggests that if two actors are similar in some way, they are more likely to have network ties; thus, OH collaborations are expected to reduce homophily by incentivizing multidisciplinary and multisectoral collaboration. Based on previous literature and our qualitative study results, we suspect the multisectoral connections may be only a temporary emergency response to address zoonosis outbreaks, such as the Avian Influenza emergency (27). SNA results also showed a greater representation of participants from human systems in zoonoses collaborations at the national and sub-national levels. Based on previously published literature on zoonoses policy process in Peru and the Mekong region, we believe this may be explained by the lack of clear roles for zoonoses management as well as conflicting governance models among agriculture, environmental, and animal health systems (21, 22, 27).

Our study is among the few that include network and qualitative data on multisectoral collaborations on zoonoses at the sub-national governmental level, addressing recommendations on the appropriate application of OH, which emphasizes the importance of incorporating local perspectives (5, 28). The sub-national level perspective highlighted the lack of OH collaboration between the national and sub-national levels, as well as the implementation challenges of cross-sectoral work. However, we also found local innovations, such as local ordinances that define roles and incentivize collaboration with external partners, including law enforcement. This is consistent with what has been found in a systematic literature review of OH programs, which proposes operationalizing and sustaining practical applications of OH at the ground level through institutional innovation, but with an established top-down OH governance (29–31).

From the interviews, we learned that national programs with isolated funding structures are barriers to resource sharing and create inequities among the sectors involved. Findings highlight barriers similar to those reported by other OH initiatives, which require unified, large-scale funding rather than disease-specific funding (29, 31, 32). Moreover, studies that evaluated OH initiatives in India and the Mekong Region that used multiple methods with zoonotic disease stakeholders found inequities in funding between animal and human systems; animal systems experienced resource limitations, whereas in some cases, human health systems reported unused funds (27, 33). From the RQM results and a report that evaluated roles and functions among organizations involved in zoonoses management, we learned that the Ministry of Agriculture has limited surveillance capacity and access to laboratories for testing infected animals (17). Moreover, consistent with similar studies on OH collaborations in Mexico, Libya, and Lao, we found that environmental systems, including national forest protection services, had limited participation and unclear roles in multisectoral zoonoses collaboration (33–35).

Mechanisms used in OH programs to address funding for joint work and coordination are found in the literature. For instance, in India, an OH steering committee independently allocates funding and resources for cross-sectoral activities (36). Similarly, in Mexico, a hierarchical, organized governance model, the General Health Council, was used to coordinate actions (13). These structures facilitate decision-making by centralizing authority in an independent party and ensuring compliance with agreements. However, there are concerns regarding the power imbalance when human health is always prioritized (33, 37). Other coordination tools focused on prioritization, process improvement, and infrastructure assessment, including the One Health Systems Mapping and Analysis Resource Toolkit and One Health Zoonotic Disease Prioritization (OHZDP) Process, have been used in various OH programs worldwide (38–40).

The application of various data collection and analysis methods in our study gave us a comprehensive understanding of OH multisectoral collaboration, including recommendations for mechanisms to fund and coordinate joint actions. We learned that it is vital to establish OH programs supported by a nationwide policy that mandates multisectoral engagement and coordination. It is also essential to engage and empower local authorities, community leaders, and farmers in the policy process to improve local surveillance systems, including the implementation of control and prevention plans for zoonotic diseases. Lastly, we recommend evaluating management tools that facilitate multisectoral collaboration and joint activities, as well as regulate roles among environmental systems in zoonosis prevention and control.

This study has some limitations. Although the RQM strengthens the study’s methodological rigor, we did not receive feedback from interested parties to validate the study’s results. We worked with local analysts who collected the data, have long worked in Peru on zoonoses prevention and control, and helped clarify questions and interpret and validate the results. We also plan to share an executive summary with the ministries involved. Snowball sampling helped to identify the management leaders of zoonoses prevention and control in Peru. Some of these individuals were high-ranking officials, such as directors or ministry heads, whose broad responsibilities and demanding schedules made them unavailable for interviews. We prioritized interviewing individuals with leadership positions and experts in zoonosis policymaking and multisectoral collaboration to address this limitation. We created an extensive list of potential participants to ensure sufficient enrolment, prioritizing the representativeness of the organizations, and interviewed at least two leaders from each organization. The sampling strategy could have influenced the homophily results by including people whose activities were focused on OH multisectoral activities. However, our focus was to give precedence to policy-making expertise and decision-making power. Lastly, SNA results provided only individual-level information on multisectoral collaborations, rather than organization-level connections. Thus, partnerships of which participants were unaware may have been missed.

Peru continues to experience several political crises, with increasing leadership turnover in government ministries. Therefore, the network data may not reflect the current state of policy action. However, our results provide recommendations for future OH initiatives. Research that evaluates OH collaborations over time and incorporates frameworks such as Adaptive Governance, which proposes integrating local knowledge into policy research and collaborating with policymakers to identify research gaps, could provide more practical and up-to-date recommendations for OH programs (41).

## 5 Conclusion

The One Health approach has been implemented to prevent and control zoonoses in low-resource countries. However, organizational and policy-related challenges affect OH implementation. Our study examined zoonoses policy networks across national and sub-national governmental levels within Peru’s human, agricultural, and environmental systems. We applied SNA to evaluate cross-sectoral collaborations and used RQM to describe mechanisms for resource management and coordination of OH multisectoral collaborations. SNA results displayed multisectoral collaborations among participants affiliated with human and agricultural systems at the national and sub-national levels, with the involvement of regional authorities and administrations. Our study also rejected the homophily hypothesis, meaning that connections related to OH collaborations were more likely among participants affiliated with different organizations. RQM results indicate barriers at the local level, such as inadequate regulation and insufficient funding for cross-sectoral activities, which create inequities in resources available to human and agricultural systems. Additionally, we identified the need to formalize functions from sectors previously not involved in zoonoses management, such as the environmental systems and forest protection services units. Future research should focus on evaluating OH policy networks over time, using frameworks that encourage local participation, particularly at the sub-national government level (14–17, 42).

## Data Availability

Supplementary material 3 provides a more detailed description of the results for each theme by organization and level of government. The qualitative datasets generated and/or analyzed during the current study are not publicly available because they could include identifiers. Requests to access the datasets should be directed to lmdumetpoma@chestnut.org.

## Author’s note

This manuscript is the result of a dissertation research project led by the first author, Lisset Dumet (LD), currently a Post-Doctoral Fellow at Chestnut Health Systems- Lighthouse Institute. The manuscript was written during her doctoral studies at Oregon Health Science University – Portland State University School of Public Health. LD was born in Peru and is currently residing in the US. All authors contributed to the manuscript. Vanessa Cruz, Ruth Atto, and Percy Vilchez, research coordinators and citizens of Peru, contributed to the data collection, analyses, and revision of the main manuscript. Erin Kenzie, Julia Goodman, Veronika Merino, and Seth O’Neal are senior researchers who advised on the main dissertation project.
