# Supplementary material for: Cross-sectoral collaborations and funding and coordination mechanisms in One Health zoonoses management in Peru
Source: Front Public Health. 2026 May 6;14:1799546. doi: 10.3389/fpubh.2026.1799546 (PMC13189141; doi:10.3389/fpubh.2026.1799546)
Supplement: Supplementary file 1 [file Data_Sheet_1.DOCX]

Figure A1: *Conceptual research framework informed by the Institutional Analysis and Development Framework, Socio-Ecological Systems and One Health* ^1^


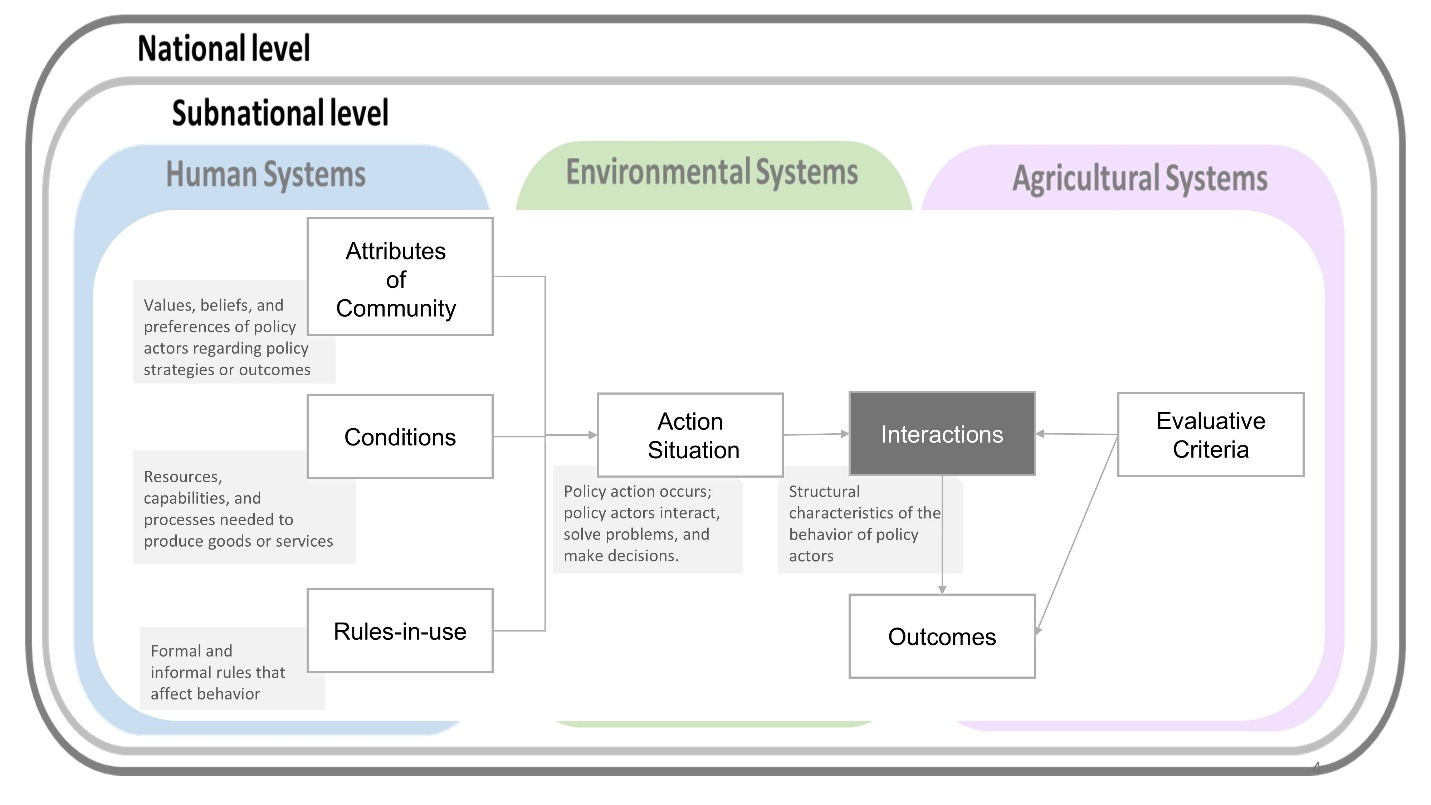


**References**

1. Dumet L, Kenzie ES, Merino V, Cruz V, Atto R, Vilchez P, et al. Applying the One Health approach to study the policy and institutional determinants to control and prevent zoonoses in a low-resource setting. Health Res Policy Sys. 2025 Nov 6;23(1):148.
