# Supplementary material for: Cross-sectoral collaborations and funding and coordination mechanisms in One Health zoonoses management in Peru
Source: Front Public Health. 2026 May 6;14:1799546. doi: 10.3389/fpubh.2026.1799546 (PMC13189141; doi:10.3389/fpubh.2026.1799546)
Supplement: Supplementary file 2 [file Data_Sheet_2.DOCX]

**Additional Document A.2. – Methods**

We used a multiple methods approach that includes social network analysis (SNA) and the rapid qualitative method (RQM) (Luke *et al.* 2018; Nevedal *et al.* 2021). We included organizations at the national and subnational levels in Peru involved in zoonotic disease prevention and control. At the national level, we included the Ministries of Health, Agriculture, and Environment, as well as educational institutions, all located in Lima, Peru. At the sub-national level, we focused on the regions of Tumbes and Piura, where our research team was based and had long-established relationships with regional and local government organizations. Ethical approval was obtained from the authors' institutions.

# Participants

Participants were national and sub-national policymakers and key informants integral to zoonosis programs in Peru from human, animal, and environmental systems. Some of the organizations that participants were affiliated with at the national level were the Ministries of Health, Agriculture, and Environment. We interview people from the Regional Health and Agriculture Directorates (Tumbes and Piura regions) at the sub-national level. Experts in zoonotic prevention and control from educational centers and research institutes, as well as international organizations nominated by others, were also included.

The sampling strategy was purposive and snowball sampling. The base sampling frame was obtained from the directory of the Department of Vector-Borne and Zoonotic Diseases of the Ministry of Health and the Directorate General of Animal Health from the Ministry of Agriculture. Informants recommended by interviewees and experts in zoonoses management identified during previous research work in the region were also included. We prioritized collecting data from the most frequently nominated people who were considered leaders in zoonoses management and multisectoral collaborations. Data collection ended when a high representation of the relevant zoonoses organizations was obtained.

# Data Collection and Measures

This study used two data collection methods: A network survey and a semi-structured interview. Both were completed in one session per participant that lasted an average of one hour. We completed 77 semi-structured interviews and 77 network surveys, and after revising for incomplete data, the final analytical sample consisted of 75 semi-structured interviews and 77 network surveys.

Data were collected from June 2023 through November 2023. Potential participants were invited to participate via email, phone calls, and official letters when requested by interviewees. Follow-up phone calls were made to schedule interviews. Consistent with local research practices, we did not offer compensation or incentives for participation. Interviews were conducted in Spanish by native Spanish speakers, as this is the primary language at the study site. The research team consisted of three interviewers who conducted most of the interviews in person; some were done by video conference call upon participant request. The sessions were recorded using a digital audio recorder or video recorder in case of a video conference call after obtaining consent from the participant. A native Spanish speaker transcribed the semi-structured interviews, and identifiers were assigned to guarantee anonymity in the data analysis process.

The network survey included questions about people whom participants regard as leaders in multisectoral collaborations and their experience collaborating with people outside of their organization (e.g., “with whom did you collaborate on the multidisciplinary or multisectoral strategy to prevent and control zoonoses that you were part of?”; “what are their name, role, and organization affiliation at that time?”). We calculated network measures to assess the prominence of individuals in a network and centrality measures such as in-degree, the count of nominations each participant received, and out-degree, the count of nominations made by a participant to another (Valente *et al.* 2008). The group's external ties trace the number of relationships among organizations. We also obtained network composition measures such as homophily, which measures the likelihood of individuals having higher opportunities to interact with others with similar traits (Kossinets and Watts 2009).

Questions from the semi-structured interview guide aimed to elicit descriptions of policy processes and multisectoral collaborations to prevent and control zoonoses.(Ministry of Agriculture and Natural Resources and Federal Democratic Republic of Ethiopia 2005; Pastrana *et al.* 2020) Topics included the ecological view of zoonosis risk factors, experience with multisectoral collaboration, and mechanisms for managing resources and accountability (“how did you participate in the multidisciplinary or multisector collaboration strategies?”; “how did the multidisciplinary or multisector collaboration strategies to prevent and control zoonoses share resources?”).

# Data Analysis

We processed the network data with R studio 4.2.2 and transferred the data to UCINET 6.759 to obtain network measures and figures (Borgatti, Everett and Freeman 2002; RStudio Team 2023). We calculated in-degree and out-degree multidisciplinary or multisectoral connections by organization and the percentage of connections within and outside organizations. We calculated homophily using the homophily test on the whole network associated with a partition using organization affiliation as a group type.

We analyzed the semi-structured interviews using the RQM. RQM proposes synthesizing the data into summaries and matrices instead of in-depth manual coding (Nevedal *et al.* 2021). The analysis team consisted of two coders (X & Y). We conducted three rounds of initial coding and discussion until both coders agreed and were comfortable splitting the remaining data and coding independently. The resulting summaries from this initial phase were condensed into merged summaries. In the next phase, the leading analyst (X) split the remaining transcripts and assigned the coder, making sure that both coders had representative groups of transcripts from all organizations. The final phase consisted of creating a matrix, which categorizes synthesized data to find patterns and identify similarities, differences, and trends among groups of participants (Averill 2002). The data were organized at the ministry and government levels. The summarized transcripts were split among coders, and each analyst reviewed the other coder's transcription summaries. Each coder did the rest of the analysis independently. The main themes were identified from the resulting matrix.

# Role of the funding source

This study was funded by the US National Institute of Health (NIH). The content is solely the responsibility of the authors and does not necessarily represent the official views of the US NIH. The funders had no role in study design, data collection and analysis, publication decisions, or manuscript preparation.

# Additional Document A.2- Semi-Structured Interview Guide – English

Date of Interview: ___/___/___

Participant ID:

Participant’s organization: _______________________

Job or position title: _______________________________

Policy problem:

a) ecological view of definition of problem and causes;

1. How does the organization where you work or the team you work with describe zoonoses and their causes?

[nudge] How would you describe the burden of zoonoses (e.g. cysticercosis) in the population of Peru?

[nudge] What are the main structural or proximal causes of their emergence and endemicity?

[nudge] Are there policy documents, state guides, or manuals that describe the problem and the need to address it?

b) perception of complexity;

2. Do you think the prevention and controlling zoonoses is a complex problem?

[nudge] Why?

[nudge] In which way?

c) identifying the need and problems

3. How does your organization or your team identify the problems that you will address or focus on?

4. [network] Who has influence in establishing and describing the problems that are needed to be addressed? Are they part of your team or do they work on a different unit?

i) Name, position or role, and unit:

ii) Name, position:______________________________________________

5. Does your organization or team use evidence-based information to support the need to create policies about zoonoses?

(If yes, ask the next question, otherwise jump to q.7).

6. How is the evidence base information collected?

[nudge] Is there a specific process to summarize evidence?

d) perception of responsibilities boundaries.

7. Do you think your organization or department has the responsibility to control and prevent zoonoses?

8. If not, who should have those responsibilities?

Policy

a) (formal) process of policy development

9. Are there policies aimed at improving the prevention and control of zoonoses in the country that you are familiar with?

(If yes, continue to next questions, if not, jump to p.11):

[nudge] How were these policies developed?

[nudge]: How were they initiated?

[nudge]: How were they negotiated?

[nudge]: How were they financed?

10. What facilitated the development of these policies?

[nudge]: Were there any policy windows that served as enablers?

[nudge]: Was there evidence that facilitated the process?

11. Are there other policies in your field that you are familiar with that you can talk about? (if yes continue, if not jump to p.14)

[nudge] How were these policies developed?

[nudge]: How were they initiated?

[nudge]: How were they negotiated?

[nudge]: How were they financed?

12. What facilitated the development of these policies?

[nudge]: Were there any policy windows that served as enablers?

[nudge]: Was there evidence that facilitated the process?

13. What do you consider are the key Zoonoses global policy documents that help inform the policy development in the country?

b) (informal) influence in consensus and decision-making;

14. What do you do if you want to accelerate a policy process or work decision related to policy?

15. [network] Who do you talk to if you want to accelerate the process and in which way do they help?

i) Name, position or role, and unit:

c) information sources for identifying solutions.

16. Does your organization use evidence-based information to identify and select solutions to control and prevent zoonoses?

(If yes, ask the next question, if no, jump to question p.18).

17. How is the evidence-based information collected?

[nudge] Is there a specific process to summarize evidence?

d) the role of the interviewee in policy development;

18. [network] What has been your role in formulating or contributing to the development of the policy agenda for zoonoses?

e) interests behind policy change or adoption

19. [network] Do you think it is important to continue working towards developing policy for controlling and preventing zoonoses such as cysticercosis?

d) external influence in policy development

20. How much influence does the international entities (e.g. WHO) have in national policy development and identifying policy solutions at your organization?

[nudge] Do you use the guidelines or request support for policy making from them?

21. How would you describe the relationship between international entities (e.g. WHO) and your organization?

Politics

a) systems influence (democracy, media, governance) on policy decisions and priorities

22. What political factors (democracy, media, governance) influence the policy agenda?

[nudge] Do they have a different influence in zoonoses policy making?

23. There is a list of zoonoses selected in the priority list to be addressed? How were they selected and by whom?

[nudge] What is the process to include a disease in the list?

[nudge] What evidence is needed?

[network] [nudge] Who takes part in the process?

b) policy environment for change

24. How do you see the future participation of your organization in advocating for prevention and control of zoonoses?

25. [network] How do you see yourself in the future participation of your organization in advocating for prevention and control of zoonoses?

c) the role of the interviewee in decision making

26. [network] What has been your role in policy decision making or priority setting for zoonoses?

d) Effectiveness, interest, and perception of the need for collaborative initiatives or programs to control and prevent zoonoses.

27. Have there been multidisciplinary collaboration strategies to prevent and control zoonoses in your organization? If response is no continue with following question, if yes jump to p.29.

28. Do you think there is a need to create multidisciplinary programs to prevent and control zooNoses?

29. Have you been part of these multidisciplinary collaboration strategies?

[nudge] In which way?

30. Have there been multisectoral collaboration (e.g., with other ministries, private or community organizations) to prevent and control zoonoses sponsored by your organization? If response is no continue with following question, if yes jump to p.32.

31. Do you think there is a need to create multisectoral programs to prevent and control zoonoses?

32. Which lessons does it provide to promote intersectoral and inter-programmatic collaboration with other sectors?

[nudge] Was there a specific policy as a result of this multisectoral collaboration program?

[nudge] Do they share common resources?

[nudge] Where there any changes in the norms and institutions of the health sector that provided a platform for collective action?

33. Are there other multisectoral programs that target other diseases or problems in your organization? If response is yes continue with following question, if no jump to p.35.

34. How was the process to develop this program?

[nudge] Was there a specific policy as a result of this multisectoral program?

[nudge]What lessons does it provide to promote intersectoral and inter-programmatic collaboration with other areas such as veterinary public health?

[nudge] Do they share common resources?

[nudge] Where there any changes in the norms and institutions of the health sector that provided a platform for collective action?

35. [network] Are you interested in forming part of multisectoral working groups for developing policy for controlling and preventing zoonoses such as cysticercosis?

g) external influence in policy decision making

36. The list of WHO priority Neglected Tropical Diseases (NTDs) includes some zoonotic diseases, how do you see the policy scene nationally based on those recommendations?

37. How much influence does the external or international institutions (e.g., WHO) and other international entities in the national priority setting?

Network Survey

39. How long have you worked in your organization? (Years, months)

40. How long have you worked in your current position/role? (Years, months)

41. Have you worked in more than one position (role) in this organization? [If yes, ask the following question; if not, jump to question p. 43]

42. What were your previous positions in this organization?

43. Who is your immediate supervisor? (Name, position)

44. Who do you supervise? (Name, position)

45. Who are the immediate people with whom you closely work with? By this, we mean people with whom you work on a daily basis or are considered to be part of your team (Name, position).

46. Within your team or unit, who has expertise in developing guidelines for zoonoses control and prevention? Which one have expertise in policy making? Which one has more influence for policy change? Which one has influence in setting the priorities?

Name, position:______________________________________________

47. Within your team or unit, who do you go to if you need to accelerate an authorization for a new guideline for control and prevention of zoonoses?

Name, position or role: ______________________________________________

48. Outside your team (but inside your organization) who do you consider have expertise in developing guidelines for zoonoses control and prevention? Which one has expertise in policy making? Which one has more influence for policy change? Which one has influence in setting priorities?

Name, position or role, unit: ______________________________________________

49. Within your organization, have you collaborated with coworkers in developing guidelines for zoonoses control and prevention? in policy making? For policy change? For setting priorities?

Name, position or role: ______________________________________________

50. Within your organization, are there any actors who served as champions for developing and pushing policy for control and prevention of zoonoses into the agenda?

[nudge] : How were they involved? What did they do?

Name, position or role, unit: ______________________________________________

51. Within your organization, which actors played a key role in shaping the NTDs policy(ies)?

[nudge]How have they participated and why them?

Name, position or role, unit: ______________________________________________

52. Within your organization, how did these actors first began to work for NTDs?

[nudge] How were they engaged?

53. Who and what helped to involve them?

Name, position or role, unit: ______________________________________________

54. Outside of your organization, who do you consider have expertise in developing guidelines for zoonoses control and prevention? Which one have expertise in policy making? Which one has more influence for policy change? Which one has influence in setting priorities?

Name, position or role, unit, organization:

55. Outside your organization, have you collaborated with people in developing guidelines for zoonoses control and prevention? in policy making? For policy change? For setting priorities?

Name, position:______________________________________________

56. Are you involved in other projects not related to control and prevention of zoonoses that require collaboration with people who work outside your organization? If yes, ask for name of project and the people who they collaborated with.

Project, name, position or role, organization:

# Semi-Structured Interview Guide – Spanish

Guía de entrevista

Nombre del participante: DNI del participante:

Organización del participante:

Título o puesto de trabajo:

Código:

Gracias por aceptar hacer esta entrevista. Las preguntas que le haremos serán relacionadas a su trabajo en la prevención y control de enfermedades zoonóticas e identificar las personas con las que usted colabora.

Hoy ………… nos encontramos en …………… con el participante ………………, quien nos ha consentido grabar esta conversación que iniciaremos ahora.

I. Preguntas de apertura:

1. ¿Cuánto tiempo lleva trabajando en este cargo?

2. ¿Qué es lo que más le gusta de las actividades que frecuentemente realiza?

3. ¿Qué tipo de actividades realiza en su puesto de trabajo relacionadas a la prevención y control de enfermedades zoonóticas?

II. Problema de política

Ahora conversaremos sobre las enfermedades zoonóticas y las causas de la enfermedad:

4. En la institución o el equipo con el que trabaja, ¿cómo definen las enfermedades zoonóticas?

Seguimiento:

• ¿Cuáles son las principales causas de su aparición y/o endemicidad?

• ¿Cómo describiría los efectos negativos que causan las enfermedades zoonóticas en la salud de la población?

5. ¿Cree que la prevención y el control de las enfermedades zoonóticas son un problema complejo para abordarlo?

Seguimiento:

• ¿Por qué?

• ¿De qué manera?

Ahora hablaremos sobre como su organización identifica los problemas de salud que abordarán:

6. ¿Cómo seleccionan o priorizan las enfermedades zoonóticas que se abordarán o centrarán?

Seguimiento:

• ¿Cuál es el proceso para incluir una enfermedad en la lista?

• ¿Qué tipo de evidencia o información se utiliza?

• ¿Cómo se recopila la información, existe un proceso específico para resumir la evidencia?

7. [red] ¿Quién participó en el último proceso de priorización de enfermedades zoonóticas?

Seguimiento:

• Nombre, cargo o función, institución, vía de comunicación (e-mail, teléfono):

• Nombre, cargo o función, institución, vía de comunicación (e-mail, teléfono):

• Nombre, cargo o función, institución, vía de comunicación (e-mail, teléfono):

III. Proceso de desarrollo de políticas

Ahora hablaremos sobre el proceso de desarrollo de políticas enfocadas en enfermedades zoonóticas.

8. ¿Cree que su institución tiene la responsabilidad de controlar y prevenir las enfermedades zoonóticas?

• NO: ¿Quién debería tener esas responsabilidades?

9. ¿Ha participado en desarrollo de políticas o regulaciones encaminadas a mejorar la prevención y el control de las enfermedades zoonóticas? ¿Cuáles?

(SI: continúe con las siguientes preguntas; NO: salte a la pregunta 11):

10. ¿Cómo se desarrollaron estas políticas?

Seguimiento:

• ¿Cómo se iniciaron? ¿Hubo oportunidades políticas como eventos nacionales que sirvieron como habilitadores para aprobar la política?

• ¿Hubo influencia interna o externa en el desarrollo de estas políticas? ¿Cuáles? Ejemplos: medios de comunicación, gobierno nacional o regional, organizaciones externas (ej: PAHO, OMS).

• ¿Se utilizó información basada en la evidencia científica o artículos científicos que facilitó el proceso?

• ¿Cómo se financiaron?

• ¿Cómo se negociaron?

• ¿Cuál ha sido su rol en la formulación?

Salte a pregunta 13 si contestó la 10

11. ¿Conoce sobre otras políticas que no estén enfocadas en enfermedades zoonóticas con las que esté familiarizado y de las que pueda opinar?

(SI: continúe, NO: salte a la pregunta 12)

Seguimiento:

• ¿Cómo se desarrollaron estas políticas?

• ¿Cómo se iniciaron? ¿Hubo oportunidades políticas como eventos nacionales que sirvieron como habilitadores para aprobar la política?

• ¿Hubo influencia interna o externa en el desarrollo de estas políticas? ¿Cuáles? Ejemplos: medios de comunicación, gobierno nacional o regional, organizaciones externas (ej: PAHO, OMS).

• ¿Se utilizó información basada en la evidencia científica o artículos científicos que facilitó el proceso?

• ¿Cómo se financiaron?

• ¿Cómo se negociaron?

• ¿Cuál ha sido su rol en la formulación?

12. ¿Si usted tuviera la oportunidad de proponer políticas o regulaciones, cuáles propondría para mejorar la prevención y el control de las enfermedades zoonóticas en la región o en el país?

Ahora hablaremos sobre el proceso de identificación de soluciones o intervenciones a los problemas relacionados a enfermedades zoonóticas.

13. ¿Cómo seleccionan las estrategias de abordaje para controlar y prevenir las zoonosis?

Seguimiento:

• ¿Alguna evidencia o información que utiliza?

• ¿Cómo se recopila la información, existe un proceso específico para resumir la evidencia?

14. ¿Cuáles son los principales documentos de políticas internacionales sobre zoonosis que influyen en el desarrollo de políticas en su institución?

IV. Política

A continuación, discutiremos los procesos de toma de decisiones en la política relacionados a enfermedades zoonóticas.

15. ¿Qué hace usted, si desea acelerar un proceso o una decisión de trabajo relacionada con una política que se propondrá?

Si la respuesta es negativa pasar a la pregunta 17

16. [red] ¿Con quién habla o se comunica si quiere acelerar el proceso o una decisión de trabajo relacionada con una política que se propondrá?

a) Nombre, cargo o función, institución, ¿Es su supervisor o jefe?:

b) Nombre, cargo o función, institución, ¿Es su supervisor o jefe?:

c) Nombre, cargo o función, institución, ¿Es su supervisor o jefe?:

17. ¿Qué factores externos o internos influyen en la toma de decisiones respecto a las enfermedades zoonóticas que se priorizarán o se atenderán? Ejemplos: medios de comunicación, gobierno central o regional, organizaciones externas (ej: PAHO, OMS).

Seguimiento:

• ¿Cuáles tienen más influencia en las enfermedades zoonóticas que se priorizarán o se atenderán?

• ¿Cómo influyen en la toma de decisiones respecto a las enfermedades zoonóticas?

18. [red] Dentro de su organización (mencionar la institución) ¿A quiénes usted consideraría líderes para desarrollar e impulsar políticas para el control y la prevención de enfermedades zoonóticas?

a) Nombre, cargo o función, vía de comunicación (e-mail, teléfono): __

19. [red] Fuera de su organización, ¿A quiénes usted consideraría líderes para desarrollar e impulsar políticas para el control y la prevención de enfermedades zoonóticas?

a) Nombre cargo o función, institución, vía de comunicación (e-mail, teléfono):

Ahora hablaremos sobre las iniciativas de colaboración multisectoriales relacionados a enfermedades zoonóticas.

20. ¿Cree que son necesarios los programas o estrategias multidisciplinarios o multisectoriales para prevenir y controlar las zoonosis?

Seguimiento:

• ¿Por qué cree que son necesarios?

21. ¿Existen estrategias de colaboración multidisciplinaria o multisectorial para prevenir y controlar las zoonosis lideradas por su institución? NO: salte a la pregunta 24.

22. ¿Ha formado parte de estas estrategias de colaboración multidisciplinaria o multisectorial?

NO: salte a la pregunta 24

Seguimiento:

• ¿Qué estrategias?

• ¿De qué manera participó?

• ¿Hubo una política específica como resultado de este programa de colaboración multisectorial?

• ¿Compartieron recursos comunes? ¿Cuáles?

• ¿Hubo cambios en las normas o reglas en la organización para incentivar colaboraciones multisectoriales o una acción colectiva?

• ¿Qué aprendizajes se obtuvieron para promover la colaboración multidisciplinaria o multisectorial?

23. [red] ¿Con quién colaboró en la estrategia multidisciplinaria o multisectorial?

a) Nombre, cargo o función, institución, vía de comunicación (e-mail, teléfono):

24. ¿Su institución ha apoyado colaboraciones multisectoriales lideradas por otras organizaciones (ej., con otros ministerios, organizaciones privadas o comunitarias) para prevenir y controlar las zoonosis? NO: salte a la pregunta 26.

Seguimiento:

• ¿De qué manera participaron?

• ¿Hubo una política específica como resultado de este programa de colaboración multisectorial?

• ¿Compartieron recursos comunes? ¿Cuáles?

• ¿Qué lecciones usted considera se obtuvieron para promover la colaboración intersectorial con otras áreas?

25. [red] ¿Con quién colaboró en la estrategia multidisciplinaria o multisectorial?

d) Nombre, cargo o función, institución, vía de comunicación (e-mail, teléfono):

26. ¿Existen otros programas multisectoriales o intersectoriales que aborden otras enfermedades que no sean enfermedades zoonóticas en su organización?

NO: salte a la pregunta 28.

27. ¿Ha formado parte de estas estrategias de colaboración multidisciplinaria?

NO: salte a la pregunta 28

Seguimiento:

• ¿De qué manera?

• ¿Hubo una política específica como resultado de este programa de colaboración multisectorial?

• ¿Compartieron recursos comunes? ¿Cuáles?

• ¿Hubo cambios en las normas o reglas en la organización para incentivar colaboraciones multisectoriales o una acción colectiva?

• ¿Qué aprendizajes considera que se obtuvieron para promover la colaboración multidisciplinaria?

28. [red] ¿Le interesaría formar parte de grupos de trabajo multisectoriales para el desarrollo de políticas de control y prevención de zoonosis como la cisticercosis?

Seguimiento:

Si la respuesta es no: ¿Por qué?

29. ¿Ha escuchado sobre el enfoque de Una Salud o One Health en inglés?

Seguimiento: ¿Qué ha escuchado?

NO: explicar concepto y saltar a la pregunta 31.

30. ¿Ha utilizado el enfoque Una Salud en su trabajo? ¿Cómo?

31. ¿Le gustaría aprender más de Una Salud para aplicarlo en su trabajo?

Muchas gracias por su tiempo hemos terminado con la entrevista.
