# Supplementary material for: Cross-sectoral collaborations and funding and coordination mechanisms in One Health zoonoses management in Peru
Source: Front Public Health. 2026 May 6;14:1799546. doi: 10.3389/fpubh.2026.1799546 (PMC13189141; doi:10.3389/fpubh.2026.1799546)
Supplement: Supplementary file 3 [file Data_Sheet_3.DOCX]

**Table A1.** Central themes regarding participants' experience in multisectoral collaboration and mechanisms for resource sharing accountability and coordination to work collaboratively in multisectoral activities by organization.

| **MULTISECTORAL COLLABORATION EXPERIENCE** | **MECHANISMS FOR RESOURCE SHARING** | **MECHANISMS FOR COORDINATION AND ACCOUNTABILITY** |
| --- | --- | --- |
| **MINISTRY OF HEALTH** |  |  |
| Most participants from the Ministry of Health (20/25 participants) reported having partaken in multisectoral collaboration to prevent and control zoonoses. | - Each organization finances multisectoral activities with its own budget. -There is funding support that comes from external organizations. - Resources are shared at the regional level. | -Political instability is a barrier to sustainability. -Multisectoral collaborations should have legal backing. -Multisectoral collaborations need Incentives for participation and inclusivity. |
| **MINISTRY OF AGRICULTURE** |  |  |
| Half of the Ministry of Agriculture’s participants (4/8 participants) reported joining a multisectoral collaboration, which resulted in a policy document. | - Emergencies such as Avian Influenza have incentivized the sharing of resources. - There is financial support from producers and external institutions. - Sharing resources is complicated. | - Multisectoral collaboration activities and their outputs need to be disseminated. - There should be a focus on preventive work. -Objectives must be clear and established in the long term.  - There is a need to clarify the roles of relevant departments and organizations. |
| **MINISTRY OF ENVIRONMENT** |  |  |
| All participants from the Ministry of Environment (2 participants) had experience with multisectoral collaborations. | - Multisectoral meetings are funded by international organizations. -Own resources finance multisectoral activities. | - One Health needs to be applied in multisectoral collaborations. - A policy with a comprehensive approach to managing zoonoses is needed. |
| **REGIONAL HEALTH DEPARTMENT** |  |  |
| Most representatives of the Regional Directorate of Health (17/20 participants) reported having participated in a multisectoral collaboration, with eight participants reporting collaborations resulting in a policy document. | - There is no specific budget for multisectoral activities, which creates duplication and waste. - Only information is shared, but there are no clear rules. - Resources are shared with local municipalities. - External organizations support multisectoral activities. | - Collaboration needs the inclusion of local government and community. - Local ordinances are effective tools for defining roles in multisectoral work. |
| **REGIONAL AGRICULTURAL DEPARTMENT** |  |  |
| Four out of five participants interviewed from the Regional Directorate of Agriculture reported having experience with multisectoral collaborations. | - Each department provides its own resources in multisectoral activities. - The organization does not have enough funding to complete multisectoral activities. - Sometimes, transportation or human resources are shared with other sectors. | - There are periodic meetings, but they need better accountability mechanisms to maintain engagement.  - There is a need for leaders to incentivize sharing of information. |
